# Heterogeneity in brain distribution of activated microglia and astrocytes in a rat ischemic model of Alzheimer’s disease after 2 years of survival

**DOI:** 10.18632/aging.103411

**Published:** 2020-06-05

**Authors:** Lidija Radenovic, Marija Nenadic, Marzena Ułamek-Kozioł, Sławomir Januszewski, Stanisław J. Czuczwar, Pavle R. Andjus, Ryszard Pluta

**Affiliations:** 1 Center for Laser Microscopy, Faculty of Biology, University of Belgrade, Belgrade, Serbia; 2 Laboratory of Ischemic and Neurodegenerative Brain Research, Mossakowski Medical Research Centre, Polish Academy of Sciences, Warsaw, Poland; 3 Department of Pathophysiology, Medical University of Lublin, Lublin, Poland

**Keywords:** brain ischemia, Alzheimer’s disease, aging, glia, neuroinflammation

## Abstract

The present study was designed to follow neuroinflammation after ischemic brain injury in the long-term survival rat model. Immunohistochemistry was performed 2 years after 10 min global brain ischemia due to cardiac arrest. For the visualization of the cellular inflammatory reaction microglial marker Iba1 and astrocyte marker GFAP were used. In post-ischemic animals our study revealed significant activation of astrocytes in all tested brain regions (hippocampal CA1 and CA3 areas and dentate gyrus, motor and somatosensory cortex, striatum and thalamus), while microglial activation was only found in CA1 and CA3 areas, and the motor cortex. In the specifically sensitive brain areas microglia and astrocytes showed simultaneously significant activation, while in the resistant brain areas only astrocytes were activated. Thus, there was clear evidence of less intensive neuroinflammation in brain areas resistant to ischemia. Such neuroinflammatory processes are backed by microglia and astrocytes activity even up to 2 years after ischemia-reperfusion brain injury. Our study thus revealed a chronic effect of global cerebral ischemia on the neuroinflammatory reaction in the rat brain even 2 years after the insult.

## INTRODUCTION

Ischemic brain injury is the second most common cause of mortality and the third cause of disability in humans [1], resulting in neurological and cognitive deficits, and eventually in dementia of Alzheimer’s disease type [2, 3]. The incidence of dementia following the first ischemic stroke is estimated in 10% of survivors and following recurrent stroke in 33-41% [4]. In long-term, 25 years follow-up of stroke-related dementias, the incidence of dementia was estimated at 48% [5]. Neurological deficits following ischemic stroke in survivors tend to progress to a different extent. On the other hand, cognitive functions gradually deteriorate leading to dementia of Alzheimer’s disease type. Presumably, in the global stroke population, the ischemia-reperfusion episodes will soon become the leading cause of death [1, 6] and of the Alzheimer’s disease type dementia [2, 3].

Recent research has shown that the ischemic brain injury could induce the neuropathology of Alzheimer’s disease type, possibly facilitating the development of dementia, due to amyloidogenesis - processing of the amyloid protein precursor into amyloid [7–9], as well as to the changes in the structure of the tau protein [10–13]. It has been documented that in the human brain, following total and focal ischemia-reperfusion episode, an accumulation of amyloid in the intra- and extracellular spaces occurs [14–17]. It has been shown that both diffuse and senile amyloid plaques form mainly in the cortex and hippocampus [14–17].

Following brain ischemia-reperfusion injury in the rat accumulation of amyloid has also been reported in the hippocampus and cortex, as well as in the white matter [18–20]. In the same animal model, amyloid deposition was observed in neuronal as well as in neuroglial cells [18–20]. The accumulation of diffuse amyloid plaques in response to ischemia-reperfusion brain injury in rats was not transient, since it has been documented that these plaques transform into senile amyloid plaques during one year after ischemic episode [21].

Post-ischemic accumulation of tau protein in neuronal and neuroglial cells was found in the hippocampus and cortex [10, 11, 13]. It has been shown that the dysfunctional tau protein may inhibit the transport of amyloid protein precursor into the cells. In animals and humans dysfunctional tau protein could form paired helical filaments leading to the development of neurofibrillary tangle-like or typical tangle structures [22–24].

Elevated amyloid levels in the post-ischemic animal brain increases the inflammatory response, infarct volume and may affect neurological outcomes [25–28]. The elevated level of soluble β-amyloid peptide predisposes neurons to both hyperactivity and excitotoxicity [29], that can be associated with an increased microglial response [30, 31]. Neuroinflammation modulates the processing of the amyloid protein precursor into amyloid by upregulating the amyloid protein precursor and β-secretase, thereby establishing a specific *vicious circle* [32–34]. Mice overexpressing the extremely aggregation-prone tau protein show activation of microglia in the brain, all leading to extensive neuronal death [35]. Another study shows that the reactive microglia causes tau protein pathology, contributing to the spread of dysfunctional tau protein in the brain, thus creating the self-perpetuating *vicious cycle* [36]. Several studies suggested that the resident inflammatory cells, microglia, are the first to respond to ischemia-reperfusion injury in the brain [25, 26, 37, 38] and that through cross-talk with astrocytes they expand neuroinflammation. The neuroinflammatory response following stroke in mice is closely related to the progress and prognosis of stroke in patients [39]. However, the exact effect of microglia on the developing neuroinflammation and its involvement in ischemic-reperfusion brain injury in humans and animals has not been investigated for the long run. With the onset of ischemic brain injury astrocytes aggressively participate in the generation of proinflammatory factors [40]. Depending on the phase of post-ischemic brain pathology, astrocytes can also show anti-inflammatory properties such as in the case of glial scar formation [41]. Notably, no studies have examined the mutual response of microglia and astrocytes in different brain regions under post-ischemic conditions, particularly upon survival time of up to 2 years, and of its translational value. Therefore, the purpose of this study was to determine if post-ischemic activity of microglia and astrocytes, 2 years after the insult, shows regional differences and whether these can be associated with previously described neuronal and functional changes.

## RESULTS

### Neuroinflammatory response in the rat brain two years after ischemia

**Figure 1:**
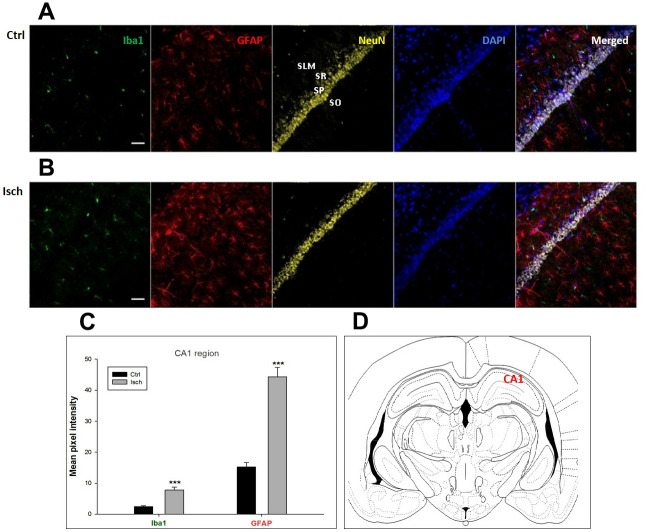
**Confocal images of microglia and astrocytes in the post-ischemic CA1 region of the rat brain.** Fourfold immunofluorescence labeling microglia with Iba1 (green), astrocytes with GFAP (red), neurons with NeuN (yellow), and nuclei with DAPI (blue). SO - stratum oriens, SP – stratum pyramidale, SR – stratum radiatum, SLM – stratum lacunosum moleculare. The scale bar represents 50 μm. (**A**) Ctrl – control brain, (**B**) Isch – post-ischemic brain, (**C**) Quantification of the mean pixel intensities for Iba1 and GFAP signals of post-ischemic *vs.* control animals with 2 years survival. Values are presented as mean ± SEM. *** p<0.001. n_Ctrl_ = 16, n_Isch_ = 17, n = number of analyzed cross sections. (**D**) Schematic representation of the rat hippocampus level with CA1 region indicated.

**Figure 2:**
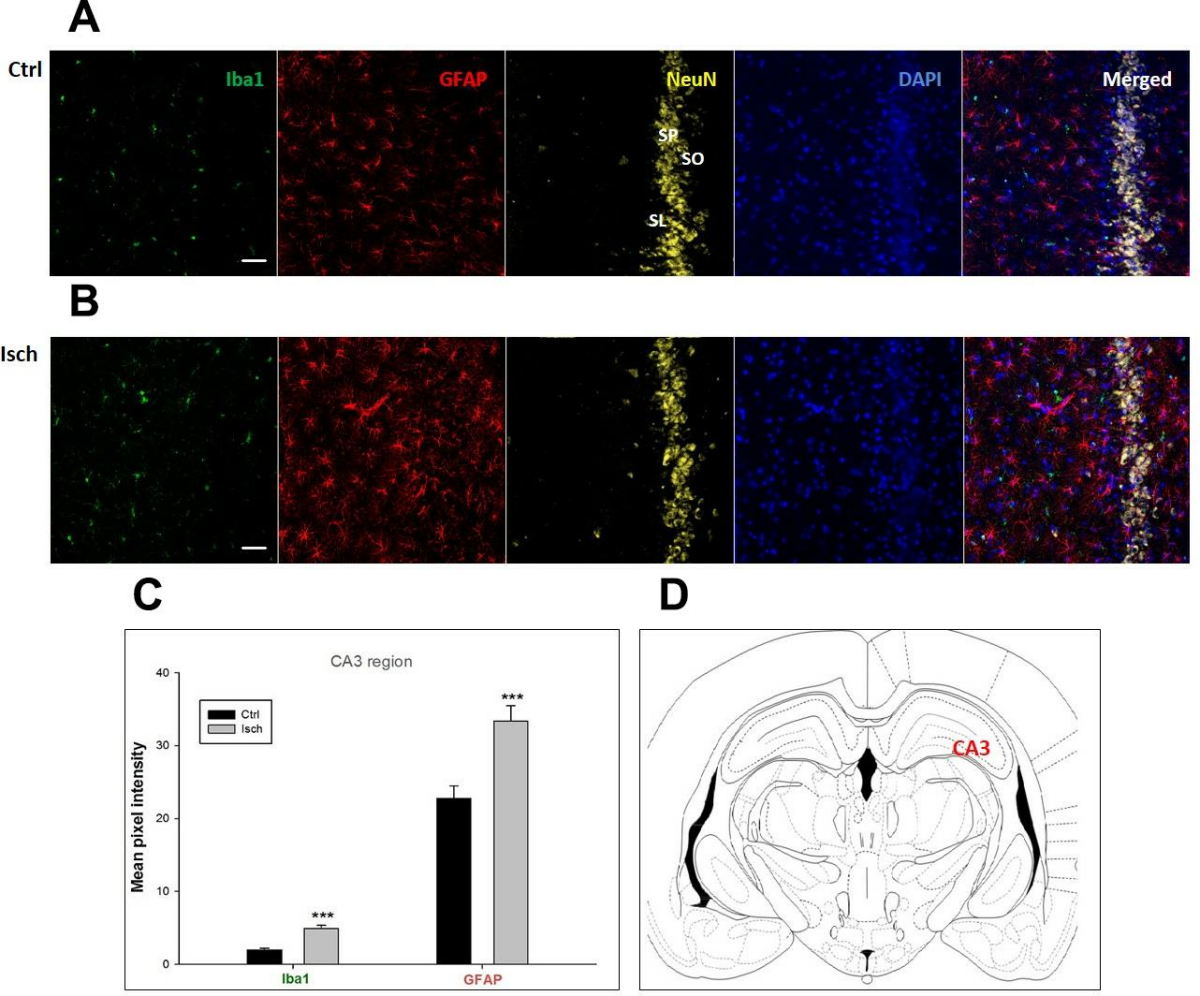
**Confocal images of microglia and astrocytes in the post-ischemic CA3 region of the rat brain.** Fourfold immunofluorescence labeling microglia with Iba1 (green), astrocytes with GFAP (red), neurons with NeuN (yellow), and nuclei with DAPI (blue). SO – stratum oriens, SP – stratum pyramidale, SL – stratum lacunosum. The scale bar represents 50 μm. (**A**) Ctrl – control brain, (**B**) Isch – post-ischemic brain, (**C**) Quantification of the mean pixel intensities for Iba1 and GFAP signals of post-ischemic *vs.* control animals with 2 years survival. Values are presented as mean ± SEM. *** p<0.001. n_Ctrl_ = 18, n_Isch_ = 18, n = number of analyzed cross sections. (**D**) Schematic representation of the rat hippocampus level with CA3 region indicated.

**Figure 3:**
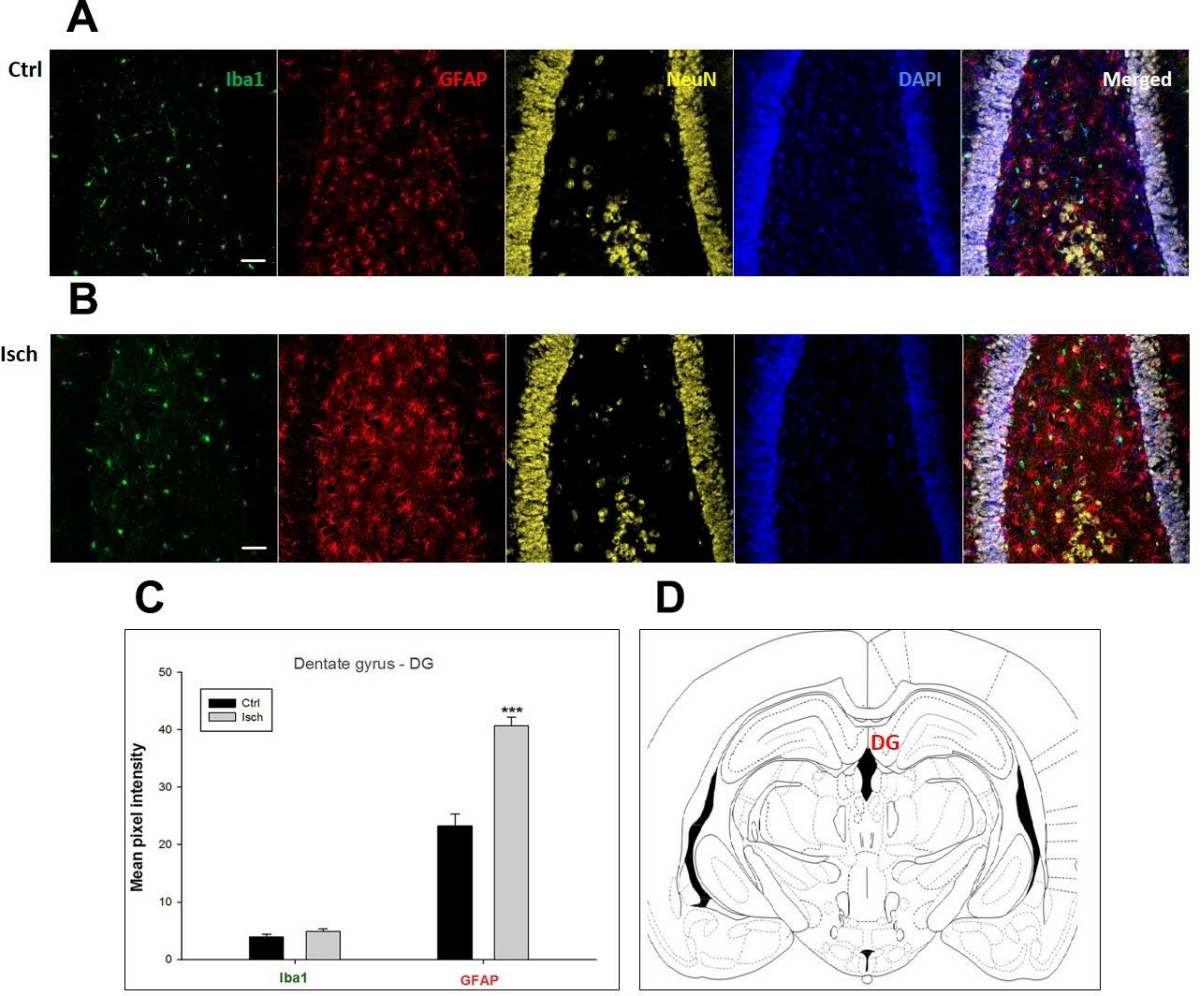
**Confocal images of microglia and astrocytes in the post-ischemic dentate gyrus (DG) of the rat brain.** Fourfold immunofluorescence labeling microglia with Iba1 (green), astrocytes with GFAP (red), neurons with NeuN (yellow), and nuclei with DAPI (blue). The scale bar represents 50 μm. (**A**) Ctrl – control brain, (**B**) Isch – post-ischemic brain, (**C**) Quantification of the mean pixel intensities for Iba1 and GFAP signals of post-ischemic *vs.* control animals with 2 years survival. Values are presented as mean ± SEM. *** p<0.001. n_Ctrl_ = 20, n_Isch_ = 20, n = number of analyzed cross sections. (**D**) Schematic representation of the rat hippocampus level with DG region indicated.

**Figure 4:**
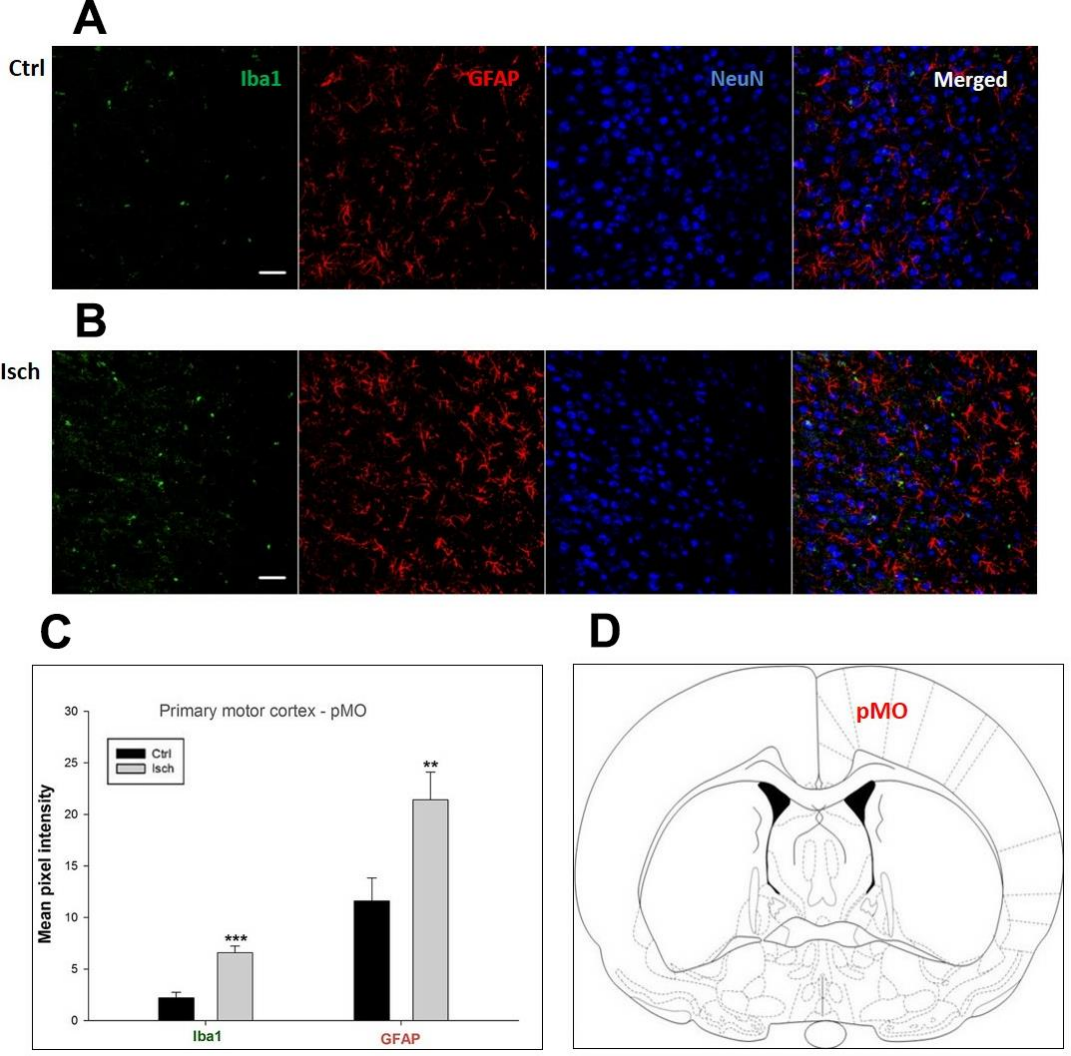
**Confocal images of microglia and astrocytes in the post-ischemic primary motor cortex (pMO) of the rat brain.** Triple immunofluorescence labeling microglia with Iba1 (green), astrocytes with GFAP (red), and neurons with NeuN (blue). The scale bar represents 50 μm. (**A**) Ctrl – control brain, (**B**) Isch – post-ischemic brain, (**C**) Quantification of the mean pixel intensities for Iba1 and GFAP signals of post-ischemic *vs.* control animals with 2 years survival. Values are presented as mean ± SEM. ** p<0.01, *** p<0.001. n_Ctrl_ = 10, n_Isch_ = 13, n = number of analyzed cross sections. (**D**) Schematic representation of the rat striatal level with pMO region indicated.

**Figure 5:**
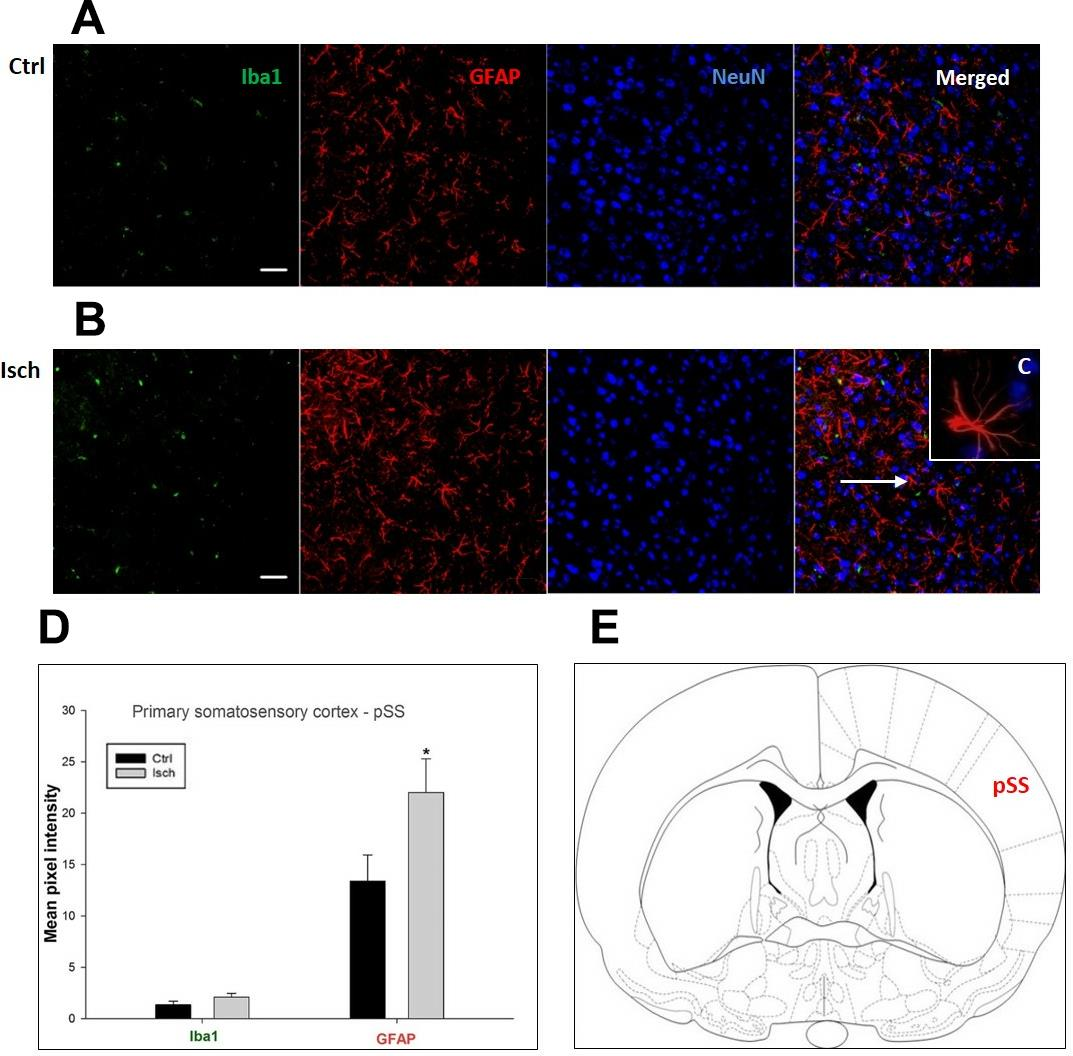
**Confocal images of microglia and astrocytes in the post-ischemic primary somatosensory cortex (pSS) of the rat brain.** Triple immunofluorescence labeling microglia with Iba1 (green), astrocytes with GFAP (red), and neurons with NeuN (blue). The scale bar represents 50 μm. (**A**) Ctrl – control brain, (**B**) Isch – post-ischemic brain, (**C**) Inset indicating an astrocyte interaction with neurons (8x), (**D**) Quantification of the mean pixel intensities for Iba1 and GFAP signals of post-ischemic *vs.* control animals with 2 years survival. Values are presented as mean ± SEM. * p<0.05. n_Ctrl_ = 11, n_Isch_ = 11, n = number of analyzed cross sections. (**E**) Schematic representation of the rat striatal level with pSS region indicated.

**Figure 6:**
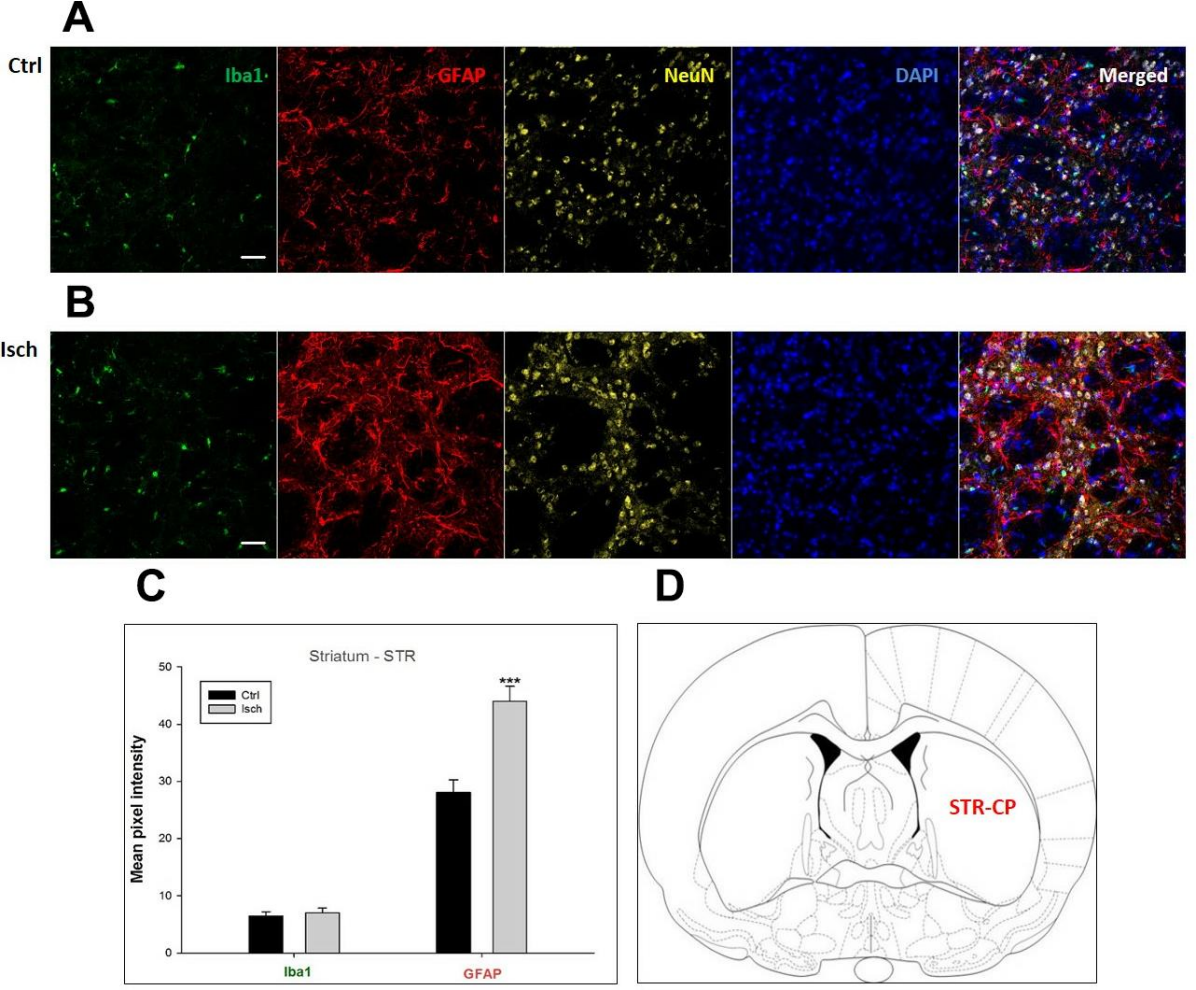
**Confocal images of microglia and astrocytes in the post-ischemic striatum-caudoputamen (STR- CP) of the rat brain.** Fourfold immunofluorescence labeling microglia with Iba1 (green), astrocytes with GFAP (red), neurons with NeuN (yellow), and nuclei with DAPI (blue). The scale bar represents 50 μm. (**A**) Ctrl – control brain, (**B**) Isch – post-ischemic brain, (**C**) Quantification of the mean pixel intensities for Iba1 and GFAP signals of post-ischemic *vs.* control animals with 2 years survival. Values are presented as mean ± SEM. *** p<0.001. n_Ctrl_ = 16, n_Isch_ = 16, n = number of analyzed cross sections. (**D**) Schematic representation at rat striatal level with STR-CP region indicated.

**Figure 7:**
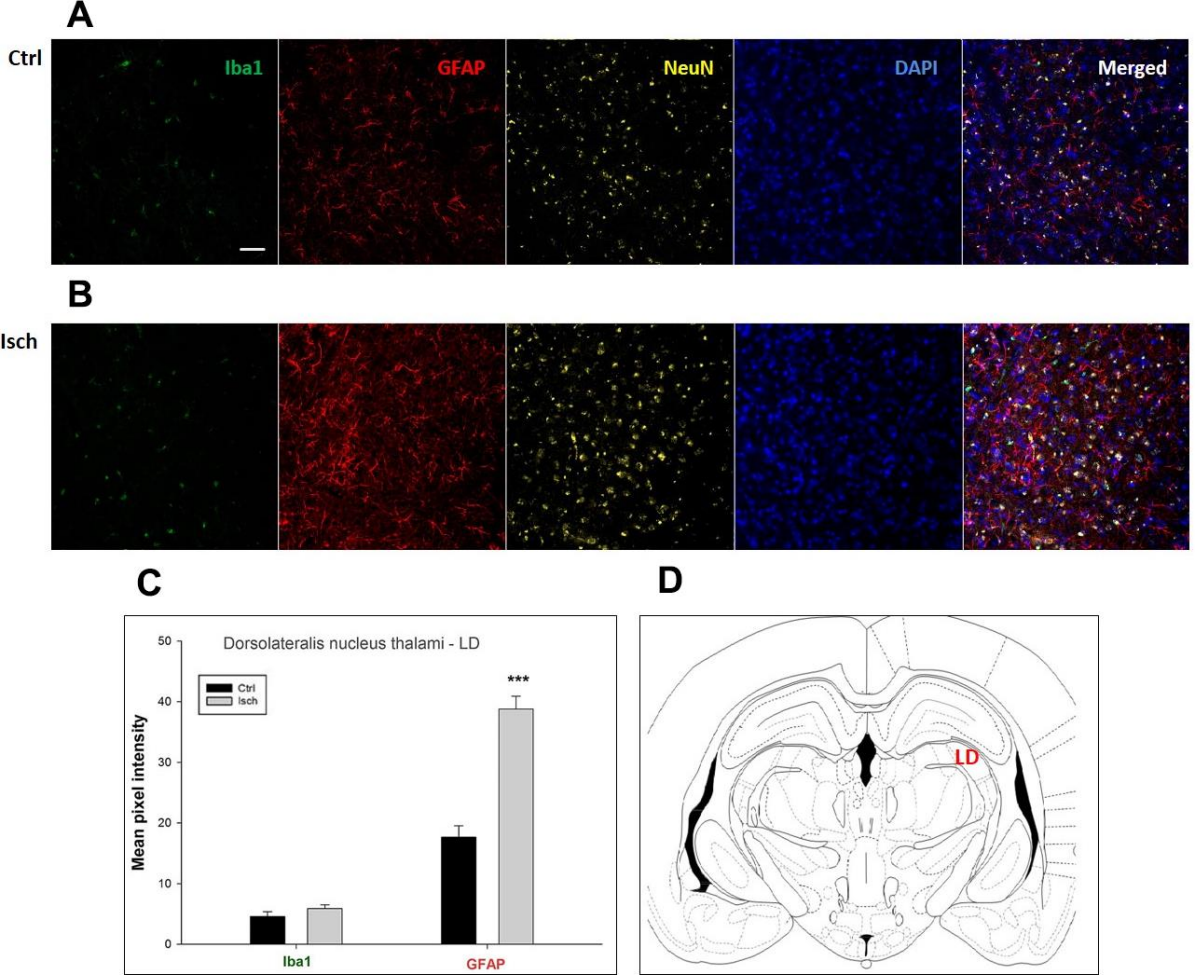
**Confocal images of microglia and astrocytes in the post-ischemic dorso-lateral nucleus of thalami (LD) of the rat brain.** Fourfold immunofluorescence labeling microglia with Iba1 (green), astrocytes with GFAP (red), neurons with NeuN (yellow), and nuclei with DAPI (blue). The scale bar represents 50 μm. (**A**) Ctrl – control brain, (**B**) Isch – post-ischemic brain, (**C**) Quantification of the mean pixel intensities for Iba1 and GFAP signals of post-ischemic *vs.* control animals with 2 years survival. Values are presented as mean ± SEM. *** p<0.001. n_Ctrl_ = 18, n_Isch_ = 18, n = number of analyzed cross sections. (**D**) Schematic representation of the rat hippocampus level with LD region indicated.

Two years post-ischemia, in 26 months old rat brain, we found in the hippocampal CA1 (Figure 1) and CA3 (Figure 2) areas, dentate gyrus (DG) (Figure 3), primary motor cortex (pMO) (Figure 4), primary sensory cortex (pSS) (Figure 5), and in striatum-caudoputamen (STR-CP) (Figure 6), as well as in the dorso-lateral nucleus of thalamus (LD) (Figure 7) a significant increase of astrocytes (GFAP-positive cells) activity in post-ischemic animals *vs.* sham controls. On the other hand, the study also showed significant microglial activation and infiltration in the rat hippocampal CA1 and CA3 regions and motor cortex (Figures 1, 2, 4). Microglia (Iba1-positive cells) of the ramified type was widespread in CA1 and CA3 areas and motor cortex as opposed to its rare appearances in sham controls (Figures 1, 2, 4).

In hippocampal CA1 and CA3 areas as well as in the motor cortex, GFAP and Iba1 staining was most prominent and significant (Figures 1, 2, 4). Qualitatively weaker signal from NeuN (Figures 1, 2, 4) indicated brain post-ischemic tissue damage in these areas. In terms of Iba1 marker, no statistical significance has been observed between the control and the post-ischemic dentate gyrus (Figure 3B, 3C), primary sensory cortex (Figure 5B, 5D), striatum-caudoputamen (Figure 6B, 6C), and dorso-lateral nucleus of thalamus (Figure 7B, 7C), however the strong GFAP staining indicated a significant increase of astrocytes activity in these brain regions.

The lack of colocalization of NeuN and DAPI signals in the striatum-caudoputamen indicated the presence of non-neuronal cells within the observed brain region. This DAPI signal may come from macrovacuoles that contribute to the sponge-like appearance of caudoputamen (Figure 6B). This indicates that during neuroinflammation after the induced ischemia macrovacuoles and caudoputamen fissures retain blood cells such as leukocytes, possibly also thrombocytes, which infiltrate the brain through the damaged blood-brain barrier [42].

### Neurodegeneration response in the rat brain two years after ischemia

**Figure 8:**
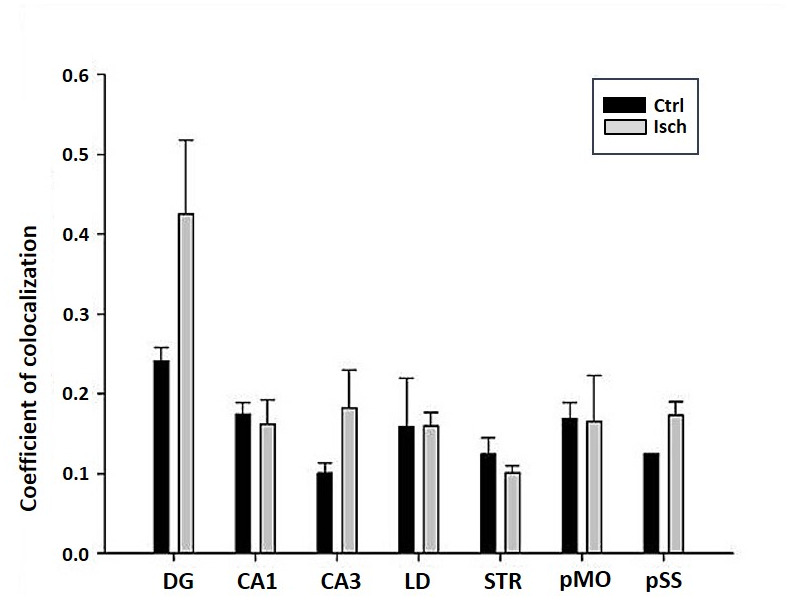
**Post-ischemic neurodegeneration of neurons in seven investigated regions of the rat brain upon 2 years of survival.** Neurons were immuno-labeled with NeuN, as a neuronal marker, and with Fluoro Jade C, as a marker of deteriorating neurons. The graph shows the quantitative analysis of the coefficient of colocalisation of the two markers in control (Ctrl) and ischemic (Isch) sections in various brain regions studied. DG - dentate gyrus, CA1 and CA3 regions of the hippocampus, LD - dorso-lateral nucleus of thalami, STR-CP - striatum-caudoputamen, pMO - primary motor cortex, pSS - primary somatosensory cortex. Data are presented as mean ± SEM.

The differences in sensitivity to ischemia among seven different brain regions was studied by following the presence of Fluoro-Jade C-labeled neurons that undergo apoptosis in post-ischemic and control brains 2 years after the insult. A trend in increased coefficient of colocalization of Fluoro Jade C and NeuN signals in post-ischemic dentate gyrus, CA3 region, and pSS was found indicating an enhanced number of neurons still entering apoptotic death even 2 years after the ischemic episode (Figure 8). However, although neurodegeneration was found in all analyzed brain regions, a significant difference in coefficient of colocalization has not been observed (Figure 8).

## DISCUSSION

We are the first to present the heterogeneity in the distribution of microglial and astrocytes activity in seven different brain structures after a 10 min ischemia in the long-term survival rat model. We observed two new microglial and astrocyte activation patterns: in the first one activation was coincidental and statistically significant in both cell types, and in the second pattern, activation was statistically significant only for astrocytes. We have thus demonstrated that in the sensitive areas associated with cerebral ischemia i.e. in the hippocampal CA1 and CA3 areas and in the motor cortex, the co-activation of both microglia and astrocytes was statistically significant. On the other hand, in the resistant brain areas, i.e. in the dentate gyrus, sensory cortex, striatum, and dorso-lateral nucleus of the thalamus, a statistically significant activation was observed only for astrocytes. In general, our study has shed more light on the diversity of microglia and astrocytes in the brain neurodegeneration phenomena after ischemia, with the simultaneous pathology shown in previous studies of amyloid and tau protein [18, 19, 43]. Differences in activation correlate with behavioral deficits associated with ischemia since these depend on the integrity of the hippocampus as well as of the motor cortex [27, 28, 44–48].

Brain ischemia causes the activation of microglia and astrocytes, which triggers the production and secretion of inflammatory mediators, i.e. cytokines [49–51]. By way of these inflammatory cytokines activated microglia and astrocytes affected post-ischemic brain pathogenesis, resulting in increased hyper-phosphorylation of tau protein, amyloid production, transcription and translation of amyloid protein precursor and tau protein in neurons, amyloid-associated pathology, decreased amyloid clearance and activation of kinases CSK-3b and CDK5 [7, 8, 10, 13, 22–24, 52–64]. CDK5 is a kinase associated with hyperphosphorylation of the tau protein [24]. Hyperphosphorylated tau protein after ischemia aggregates into paired helical filaments [23] that eventually form neurofibrillary tangles [22, 24]. During pathological conditions astrocytes begin to express β-secretase, thus obtaining the ability to produce amyloid [32–34]. Neuroinflammation mediates the synergy between brain ischemia and amyloid, causing synaptic depression [65]. It is suggested that the elevated level of soluble β-amyloid peptide predisposes neurons to both hyperactivity and excitotoxicity [29], which is associated with an increase in focal microglial response [30, 31]. The neuroinflammation seen in post-ischemic brain injury presumably appears to play a leading role in increasing the amyloid burden and tau protein dysfunction, suggesting that this dual role may be the leading link between these seemingly different features of Alzheimer’s disease pathology [53]. On the other hand, mice overexpressing mutant tau protein with high aggregation ability demonstrated stimulated hyperactivation of microglia, as well as an extensive loss of neurons that could be dampened by immunosuppression [35]. Activation of microglia contributes to the spread of dysfunctional tau protein in the hippocampus and the motor cortex. It has been mechanistically assumed that microglia phagocytes the tau protein and releases it together with exosomes, thus contributing to the spread of the dysfunctional tau protein [36]. The continuous release of proinflammatory cytokines and neurotoxins from astrocytes and microglia serves to exacerbate the inflammation of the nervous system and contribute to neurodegeneration, leading to further activation of microglia and astrocytes [66].

Chronic opening of the blood-brain barrier has been shown in our model of ischemic rats with long-term survival also confirming that the hippocampus is most susceptible to neuroinflammation and accumulation of the β-amyloid peptide [19, 26, 67]. It has been shown that the blood-brain barrier is disrupted during the brain ischemia-reperfusion injury, in which neurovascular inflammation, characterized by an up-regulation of inflammatory mediators and proteases originating from endothelial and immune cells, plays a significant role [68]. Blood-brain barrier breach may also be strongly associated with the activation of microglia [68]. It has been shown that after an ischemic stroke, the blood-brain barrier integrity was influenced by microglia *via* an up-regulation of pro-inflammatory cytokines including IL-1b, TNF-a, and IL-6 [68]. It has also been reported that under stroke conditions, brain vessels become permissive to blood serum components, which leak into the parenchyma and thus promote microglial recruitment [69]. Post-ischemic blood-brain barrier disruption may occur by several mechanisms, including the physical alterations of astrocyte-endothelial junctions [41]. Digestion of blood-brain barrier matrix proteins by astrocyte matrix metalloprotease 2 and other matrix metalloproteases contributes to the physical disruption of the blood-brain barrier [41]. Thus, functional integrity of astrocytes proves to be essential for maintaining the blood-brain barrier integrity following the ischemia-reperfusion episode, particularly by reestablishing the astrocytic water channels, AQP4, which are essential for blood-brain barrier repair during post-stroke recovery [41]. Post-ischemic hippocampal and motor cortex neurons have shown the largest loss [19, 43], which correlates here with a significant inflammatory response of microglia and astrocytes in the same brain structures. Our previous study provides evidence of the role of neuroinflammation in post-ischemic cognitive impairments that are correlated with the atrophy of the hippocampus [26, 43, 70]. Microglia and astrocytes are strongly associated with inflammatory changes in the hippocampus and likely contribute to its neurodegeneration and related deterioration of cognitive functions [27, 28, 31]. In fact, some studies suggest that changes in the hippocampus may lead to persistent deterioration of memory in humans and animals, considered as the usual consequence of brain ischemia [44, 48, 71, 72]. In studies analyzing the post-mortem volume of the hippocampus in patients with dementia after ischemia, a decrease in the volume of CA1 and CA3 areas of the hippocampus was demonstrated by approximately 20% in each of the analyzed regions [71, 72]. Some studies, including ours, have shown that the appearance of characteristic features of neurodegeneration, amyloid, and tau protein dysfunction together with inflammatory changes, closely correlated with slow cognitive impairment and dementia after brain ischemia [28, 44].

The neuronal overproduction of the β-amyloid peptide, after cerebral ischemia, stimulates astrocytes to release complement C3, which binds to C3a receptors on neurons and microglia and causes impaired phagocytosis of microglia [73]. On the other hand, the cross-talk of astrocytes with microglia through the activation of complement affects the amyloid pathology [74]. During the development of inflammatory changes after cerebral ischemia, astrocytes either release inflammatory mediators [51], or communicate directly with microglia and/or neurons to modulate the inflammatory response. In focal cerebral ischemia, astrocytes and microglia show proliferative changes in the penumbra [75], indicating that both types of neuroglial cells are activated. Activated microglia has a huge impact on astrogliosis after brain damage, by affecting the development of the glial scar [76]. The glial scar acts as a barrier that prevents axonal ingrowth and reinervation, thus hindering regeneration [50]. It is thus possible that in the ischemic hippocampus, microglia contributes to the pathological changes not only through its direct action, but also indirectly by affecting astrocytes [77]. In fact, it was confirmed that the neurotoxicity of reactive astrocytes was induced by active microglia [77]. Astrocytes and microglia also cooperate in the phagocytosis of ectopic neurons [78]. In chronic, incomplete brain ischemia in rats, neurons with microglia and astrocytes work together creating ectopic and apoptotic neurons, as well as residual neurons. Astrocytes processes can then penetrate into the bodies of ectopic neurons, creating triads with activated microglia [78]. The formation of triads intensifies ischemic processes and leads to severe neurodegeneration. Throughout the progress of the pathological processes, astrocytes on the other hand, may also affect microglia by inhibiting its activity.

Our present study demonstrates that the neuroinflammatory response in the ischemic CA1 and CA3 areas and motor cortex clearly confirms a chronic character with powerful destructive influence. These findings reveal that the active post-ischemic neuroglial response in the hippocampus and the motor cortex lasts much longer than initially thought [79, 80] coinciding with the development of severe and progressive neurodegeneration and dementia after ischemia [28, 44, 46–48].

On the other hand, our study also shows that in ischemic areas of the brain, such as the dentate gyrus of the hippocampus, the sensory cortex, the striatum and the dorso-lateral nucleus of the thalamus, astrocytes alone may also play a role in the progression of cerebral ischemia. Previous studies in this brain ischemia model have shown activation of astrocytes with overexpressed cytokines IL-1β or IL-6 [51]. In the above tested structures under inflammatory changes, the effect of microglia and astrocytes on ischemic pathology is probably limited. We have in fact, shown a smaller effect of neuroinflammatory changes in resistant areas of the brain to ischemia.

Previous studies have been focused on the relationship between ischemic neurons and amyloid and tau protein pathology [7, 8, 10, 13, 18–24, 54–64] while neuroglial pathology has often been neglected. With the emerging new evidence, including the results of our study, it becomes evident that microglia and astrocytes are not only witnesses, but active and important participants in neurodegeneration in the post-ischemic brain, as well as in Alzheimer’s disease. Thus, we can foresee that the understanding of the relationship between neurons and neuroglial cells after brain ischemia will become more important in the future.

## CONCLUSIONS

Our study, revealed the role of neuroinflammation in neurodegeneration throughout the post-ischemic brain. The role is quite complex and goes beyond the scope of only one research article. These findings show that the effect of brain ischemia on the activity of microglia and astrocytes is significantly different among the brain structures studied. This partly explains why in rats the extent to which neurodegeneration occurs in the brain after ischemia varies greatly depending on the area and does not develop at the same time [7–10, 13, 81–83].

Currently, the full temporal and spatial dynamics of the inflammatory response after ischemia is unknown. One way to broaden the scope of research is to move away from the biochemical theory of post-ischemic neurodegeneration towards cellular theory instead. We hope that the new experimental approaches, such as the study of gene expression changes in neuroglial cells in our model, will provide new knowledge about the complex spatial and temporal nature of the neuroinflammatory response after brain injury as a result of ischemia and reperfusion. Finally, the development of novel cell imaging tools *in vivo* can facilitate the development of new strategies for the treatment of stroke patients.

## MATERIALS AND METHODS

### Animals

Two-month-old, female rats (Wistar, 160–180 g, n=16) were used for the study. Groups of four animals per cage, were housed in an air-conditioned room, at the temperature of 22 ± 2 °C, with 55 ± 5% humidity, and with lights 12 h/day (07.00 - 19.00). The animals were given commercial food and tap water *ad libitum.* All experimental procedures were performed during the light phase, between 9:00 and 15:00 under identical conditions. Animals used for procedures were treated in strict accordance with the European Communities Council Directive (86/609/EEC and 2010/63/EU) and with the approval of the local Ethical Committee. All efforts were made to minimize animal suffering and to reduce the number of animals used, in accordance with principles of good laboratory practice.

### Brain ischemia model in rats with long-term survival

Our animal model of global cerebral ischemia clinically represents reversible cardiac arrest. Global cerebral ischemia was performed by cardiac arrest of 10 min duration [13, 84]. The animals were allowed to survive 2 years post-ischemia. Sham-operated rats were exposed to the same procedures as ischemic animals but without induced cardiac arrest and thus served as controls.

### Immunocytochemistry

Immunocytochemistry was performed 2 years after the ischemic insult on 6 ischemic and 6 control rats. There was no mortality within 2 years after successful resuscitation (n = 6), but during cardiac arrest it reached 40% (n = 4/10). During brain autopsy of rats that died during cardiac arrest, no macroscopic lesions were observed. After transcardiac perfusion with 4 % paraformaldehyde the brains of resuscitated or sham controlled animals were postfixed in the same solution and cryoprotected in 30% sucrose. After freezing at -80^o^C brains were cut on a cryostat in 30 μm-thick coronal slices. Microglial cells were labeled with anti-Iba1 (1:250, Abcam), astrocytes with anti-GFAP (1:300, Daco), and neurons with anti-NeuN (1:100, Milipore) primary antibodies and visualized with the use of Alexa Fluor 488-conjugated donkey polyclonal anti-goat antibodies, Alexa Fluor 555-conjugated donkey polyclonal anti-rabbit, and Cy5 633-conjugated donkey polyclonal anti-mouse secondary antibodies, respectively (all 1:200, Molecular Probes). The slides were then washed with PBS and stained with the nuclear marker 4,6-diamidino-2-phenylindole (DAPI, 1:200, Molecular Probes). In parallel, double staining with anti-NeuN (1:100, Milipore) and Fluoro Jade C (1:100, Millipore) was used for visualization of neurodegenerated neurons. After washing the slides were dried and coverslipped using mounting medium (Mowiol, Sigma Aldrich). As a negative control primary antibodies were omitted. Studies of immunohistochemical reactions of different sections from all experimental and control groups were processed in parallel.

### Image acquisition

Immunostained sections were imaged by a confocal laser scanning microscope (LSM 510, Carl Zeiss GmbH) with an argon laser (488 nm) utilized for the excitation of Alexa Fluor 488 and Fluoro Jade C, and helium-neon lasers (543 nm and 633 nm), for the excitation of Alexa Fluor 555 and Cy5, respectively. Objectives used were Plan - Neofluar 20x/0.5. Laser intensities, pinhole, scan speed, digital gain and offset were maintained constant throughout imaging.

### Image analysis and quantification

Following acquisition with Zeiss LSM 510 confocal, images were processed using the Zeiss LSM 510 Basic software package v. 3.2. In order to investigate the activity of astrocytes and microglia within the specific brain regions, mean signal intensity of the Iba1 and the GFAP signal pixels were calculated for each image after thresholding for background fluorescence (six animals/group, 4–5 images/animal, in total 24-30 images/group). The number of analyzed brain slices was n=16-20 per group (control and ischemia).

In addition, we quantified by pixel analysis the colocalization of the green (Fluoro Jade C) over red (NeuN) signal. This was performed with confocal images taken from brain slices with the objective magnification of 20x, n=20 for each group (control or ischemia).

### Statistics

Experimental values were statistically compared with the Student’s t-test using Sigma Plot 11.0 software package (Systat Software, Inc.).
